# Carbon dots for photothermal applications

**DOI:** 10.3389/fchem.2022.1023602

**Published:** 2022-10-12

**Authors:** Salar Balou, Pooja Shandilya, Aashish Priye

**Affiliations:** ^1^ Department of Chemical and Environmental Engineering, University of Cincinnati, Cincinnati, OH, United States; ^2^ School of Advanced Chemical Sciences, Shoolini University, Solan, Himachal Pradesh, India

**Keywords:** photothermal, carbon dots, photothermal therapy, solar water evaporation, antibacterial activity, photothermal shape memory polymers, photothermal plastics

## Abstract

Carbon dots are zero-dimensional nanomaterials that have garnered significant research interest due to their distinct optical properties, biocompatibility, low fabrication cost, and eco-friendliness. Recently, their light-to-heat conversion ability has led to several novel photothermal applications. In this minireview, we categorize and describe the photothermal application of carbon dots along with methods incorporated to enhance their photothermal efficiency. We also discuss the possible mechanisms by which the photothermal effect is realized in these carbon-based nanoparticles. Taken together, we hope to provide a comprehensive landscape highlighting several promising research directions for using carbon dots for photothermal applications.

## Graphical Abstract

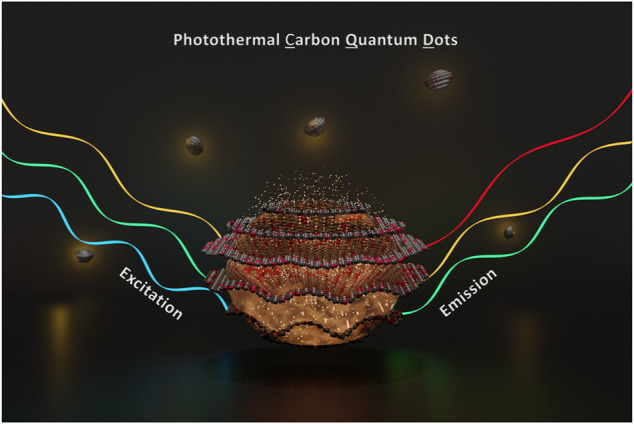

## Introduction

Carbon dots (CDs) are zero-dimensional carbon nanoparticles that can be categorized into two broad groups based on their structure: 1) graphene quantum dots—primarily composed of sp^2^-hybridized carbon (zero-dimensional graphene lattice), and 2) amorphous quantum dots–comprising of sp^3^ and sp^2^ quasi-spherical carbon nanoparticles with abundant surface functional groups (Wareing et al., 2021). Over the past decade, CDs, received tremendous attention because of their diverse merits such as simple fabrication, adaptable physical and chemical structure, biocompatibility, eco-friendly nature, water solubility, photostability, and highly tunable fluorescence emission (Liu et al., 2020). Consequently, they have routinely been applied in optical (sensing, bioimaging, and anticounterfeiting) (Li et al., 2021a), biomedical (drug/gene delivery, photodynamic therapy) (Chung et al., 2020; Jia et al., 2020), and energy (photocatalysis, supercapacitors, and light-emitting diodes) (Semeniuk et al., 2019) related applications. Additionally, these CDs have recently been harnessed to convert light to thermal energy *via* photothermal conversion (Li et al., 2021a). CDs have abundant loosely held electrons in their ground state, which, upon exposure to the proper wavelength of light, are excited to higher energy states or the excited singlet state. These CDs are well known for the radiative relaxation of their excited electrons resulting in fluorescence emission. On the contrary, the nonradiative relaxation can cause significant molecular vibrations in the nanoscale because of electron-phonon couplings, interpreted as significant heat generation in the macroscopic scale (Ye and Li, 2022). Thus, CDs can serve as an alternative to metallic-based, semiconductor, and polymer-based photothermal nanomaterials.

Here, we comprehensively summarize the role of CDs in all photothermal systems, including photothermal therapy, solar water evaporation, antibacterial activity, shape change polymers, and self-healing of coatings. We highlight the primary methods to enhance the Photothermal Conversion Efficiency (PCE) of CDs, such as metal and metalloid doping (Tejwan et al., 2021), heteroatom doping and surface modification (Ding et al., 2020), hyperconjugation, coating and hybridization, and band-gap engineering. Although CDs are considered the next generation of cost-effective, versatile, and environmental-friendly carbon-based photothermal nanomaterials (Tejwan et al., 2020), our understanding of their photothermal conversion mechanism is incomplete, which makes this minireview an important step forward toward the fundamental and practical enhancement of their PCE, to broaden their functionality towards photothermal applications.

## Photothermal applications

### Photothermal therapy

Photothermal therapy (PTT) involves the use of electromagnetic radiation to treat medical conditions like cancer *in lieu* of traditional treatment approaches like chemotherapy that exhibit major side effects. In traditional photothermal therapy, heat is generated by localized surface plasmonic resonance (LSPR) of metallic nanoparticles such as gold and silver. Here a NIR light source penetrates the skin and initiates LSPR-induced temperature rise above 40°C to dilate blood vessels and ablate cancer cells. The lack of sufficient biocompatibility with metallic nanoparticles has led to the search for non-toxic and more functional alternatives. As a result, Wang et al., reported the first successful application of CDs for photothermal cancer therapy. These CDs were encapsulated in a porous carbon matrix with Fe_2_O_3_ nanocrystals clustered in its core. The hybrid nanomaterial demonstrated combined features of magnetic resonance imaging (MRI) contrasting ability, fluorescence cell imaging, high drug loading capacity and photothermal conversion ability (Wang et al., 2014a; Wang et al., 2014b). Since then, significant efforts have been made to implement CDs as single nanoplatforms for both bio-imaging and photothermal therapy applications by simultaneous enhancement of fluorescence, PCE, and biocompatibility of CDs.

Coating of nanoparticles with inert polymers such as polyethylene glycol (PEG) has been shown to increase their biocompatibility (Havrdova et al., 2016). However, trapping CDs into the core of PEG can not only make them more biocompatible but also promote their PCE (Tu et al., 2014; Zheng et al., 2016; Lan et al., 2018). Interestingly, Hou et al. showed that PEG coating causes the red-shift of the fluorescence emission of CDs. These red emissive carbon dot assemblies demonstrated a higher PCE for *in vitro* eradication of cancer cells than their uncoated individual counterparts (Hou et al., 2017). Additionally, specific chemical and physical modifications narrow the HOMO-LUMO band gap, redshifting the fluorescence emission peak and improving their photothermal performance (Masha and Samuel Oluwafemi, 2022). Consequently, Ge et al. studied the performance of red-light emitting CDs for the *in vivo* and *in vitro* dual photodynamic and photothermal treatment of cancer. They showed simultaneous reactive oxygen species (ROS) generation and efficient photothermal conversion of CDs (Ge et al., 2016). Zhao et al. reported the lysosome-targetable yellow emissive CDs with high ROS generation and a significantly superior PEC of 73.5% applied for synergistic photodynamic therapy (PDT) and PTT (Zhao et al., 2020). They later discovered that narrower HOMO-LUMO band gaps with more interim energy bands and lattice vibrations in CDs layered graphite structures enhance the rate of nonradiative relaxations, increasing their PCE (Zhao et al., 2021).

**FIGURE 1:**
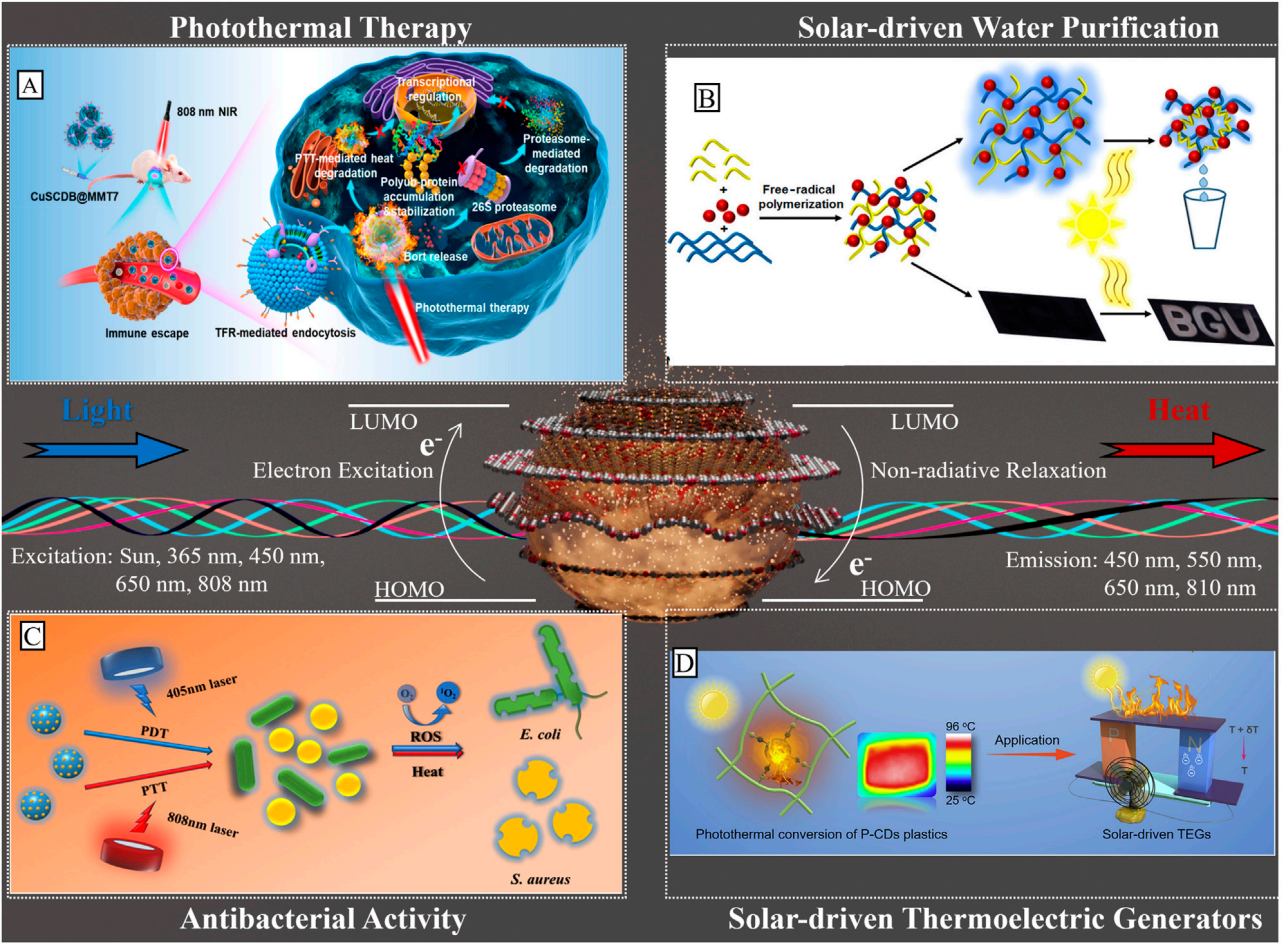
A representation of a carbon dot consisting of six disk-shaped graphite layers with both sp2-and-sp3 hybridized carbon atoms (silver spheres) beside the embedded pyridinic and pyrrolic nitrogen groups (red spheres), and the fluorescence and heat generation upon excitation with different wavelengths of light. The photothermal conversion of carbon quantum dots has been implemented in: **(A)** the photothermal degradation of a tumor cell via the CuS/Carbon Dots nanocomposites under the excitation of a 808 nm laser reprinted with permission from Yu et al. (2020), copyright 2020 American Chemical Society, **(B)** the solar water purification and optical switching with C-Dot—CMC—PNIPAMhydrogels under the sunlight illumination reprinted with permission from Singh and Jelinek (2020), copyright 2020 American Chemical Society, **(C)** Antibacterial activity of CDs/Cur system under a combined 405, and d 808 nm laser irradiation reprinted with permission from Yan et al. (2021), copyright 2021 American Chemical Society, and (D) Solar-driven thermal electric generation made possible by plastics integrated with carbon dots (P-CDs) under the sunlight irradiation, with permission from Wan et al. (2022), copyright 2022 Elsevier..

Additionally, Metal and metalloid doping of CDs has shown promising enhancements in their overall fluorescence and PCE (Zhang et al., 2017). Consequently, Gadolinium doped amino and carboxylic coated CDs have been used as nanoplatforms with both controlled drug release and fluorescence visualization owing to their high NIR induced PCE and enhanced fluorescence emission performance, respectively (Zhang et al., 2017). Moreover, doping CDs with copper has been shown to increase their PCE. The copper-doped CDs have been used for triple-modal (fluorescence, photothermal, and photoacoustic) imaging-guided phototherapy of cancer cells and laser-triggered drug delivery with minimal side effects after being coated with thiol-PEG (Bao et al., 2018). Lan et al. (2017) co-doped CDs with Selenium to generate an excitation wavelength-independent NIR emission in CDs which enabled the two-photon excitation (TPE) fluorescence and efficient *in vivo* and *in vitro* photothermal therapy of cancer cells. Guo et al. (2018) engineered a Cu-N-carbon dot complex with enhanced PCE due to the generation of N-Cu-N complex coordinates that enhances NIR adsorption. Likewise, Yu et al. prepared hollow-structured CuS composite CDs as a synergistic anticancer nanocarrier system (Figure 1A). They demonstrated a high PCE with the ability to ablate targeted tumor cells (Yu et al., 2020). Hu et al. covalently bonded Pt (IV) with red emissive CDs and formed a complex nanocarriers system with both NIR-induced photothermal effect and targeted chemotherapy. Their multifunctional nano-complex was further coated with PEG, forming a nanoprobe capable of imaging-guided chemo/photothermal elimination of cancer cells (Hu et al., 2021).

Apart from metal doping, heteroatom doping of CDs with nitrogen and oxygen-containing functional groups are among the well-known methods of enhancing their fluorescence emission (Yan et al., 2018). However, the impact of N, and O doping on the photothermal performance of CDs has been relatively less investigated. Bai et al. studied the effect of nitrogen doping (with polydopamine) on the photothermal performance of CDs for the first time, and their results indicated that N-doping can enhance the quantum yield and the PCE of CDs simultaneously (Bai et al., 2018). Generally, N-doped CDs often demonstrate NIR fluorescence emissions and NIR-induced photothermal effects. The addition of these characteristics to the excellent photostability, easy surface modification, and low toxicity of N-doped CDs makes these nanoparticles suitable for *in vitro* and *in vivo* image-guided chemo/photothermal therapies (Li et al., 2021b; Shu et al., 2021; Duan et al., 2022; Liu et al., 2022). Geng et al. showed that nitrogen dopants introduce deep defect states into the wide HOMO-LUMO band-gaps of CDs by infusing excess electrons into their π-π* orbitals, lowering the band-gap energy and increasing their PCE for tumor ablation (Geng et al., 2020). Recently, Han et al. (2022) discovered that surface modifications of CDs with negatively charged functional groups such as carboxylic and amino groups also enhance the tumor vascular permeability, thus increasing the overall efficiency of the PTT.

Metal-organic frameworks (MOFs) can be crosslinked with CDs to yield nanomaterials with high PCE for PTT and accelerated redox kinetics for PDT. Bai et al. fabricated NIR emission carbon dot-metal organic framework assemblies [MIL-100 (Fe)]. They revealed that carbon dot-induced photothermal conversion can accelerate the redox reaction generated by MOFs, which ultimately improves the thermal sensitivity of cancer cells (Bai et al., 2022). Similarly, Sun et al. (2022) showed that crosslinking amino-functionalized CDs with organometallic compounds [ferrocene (Fc)] can increase the size, enforce the selectivity toward cancer cells, and enhance the overall optical and photothermal effect of carbon dot-MOFs systems as a safe imaging-guided antitumor-specific nanoplatform. Sun et al. (2019) demonstrated that the loading of chlorin e6 as a photosensitizer onto N-doped red emissive CDs resulted in their superior photothermal performance and much more efficient noninvasive and stimuli-responsive cancer therapy at reduced laser powers.

The biocompatibility, sustainability and fabrication cost of CDs for photothermal applications can be greatly enhanced by substituting the chemical precursors with biomass and waste-derived ones at the fabrication stage (Wareing et al., 2021). Jia et al. fabricated biomass-derived red-light emitting CDs with excellent 1O_2_ and heat generation under NIR light for the synergistic photodynamic/photothermal tumor therapy. Their work showed the possibility of producing multifunctional CDs from biomass and justified the need to develop biomass-derived photothermal CDs (Jia et al., 2018). Li et al. (2019) showed that watermelon-derived CDs have NIR-II emissions (1,000–1,700 nm) beside an excellent PCE for *in vivo* ablation of cancer cells. Kim et al. fabricated sulfur-doped CDs from Camellia japonica flowers with intense NIR absorption. They demonstrated a complete tumor ablation at low laser powers due to their high PCE (Kim et al., 2021).

Meanwhile, incorporating CDs into meso/microporous structures such as porous carbon and silica framework became the topic of interest. For instance, CDs have been encapsulated into the chitosan matrix as a porous natural compound, integrating the functionalities of the former with the excellent stability and high porosity of the latter. The multifunctional nanocarrier has been used for combined chemo-photothermal treatments through a light-induced release of the preloaded drug (Wang et al., 2017). Similarly, polymer-coated CDs were uniformly incorporated into the silica framework through hydrogen/electrostatic assisted assemblies that demonstrated an enhanced PCE and were applied for the immune-mediated inhibition of tumor metastasis (Qian et al., 2019).

### Solar driven water evaporation

Water scarcity and depletion of natural freshwater resources are among the most significant environmental challenges that humanity is currently facing. Several technologies have been applied to address water purification issues, such as reverse osmosis and thermal evaporation. Recently, photothermal nanomaterials have been harnessed for solar-driven water evaporation. Among the available photothermal nanomaterials for solar to heat conversion, CDs promise high PCE and broad absorption spectra while also exhibiting low thermal conductivity conducive of low thermal dissipation and high localized heat generation (Zhou et al., 2022). The purification of contaminated water is realized through the adsorption of water in porous media with infused CDs, followed by water evaporation *via* the heat generated from the photothermal effect of CDs. Hou et al. (2019) demonstrated that *in situ* self-assembly of CDs on the surface of processed wood can develop a photothermal system with high porosity and abundant oxygen functional groups (hydroxyl groups) that are known for enhancing the solar-to-vapor conversion efficiency. Singh et al. (2019) encapsulated CDs within a porous biomass-derived hydrogel (chitosan) and fabricated a stable and recyclable nanocomposite capable of solar-mediated water evaporation and water desalination. They also demonstrated that substituting chitosan with a photothermal phase transition hydrogel can create thermally actuated optical switches in addition to water purification system (Singh and Jelinek, 2020) (Figure 1B).

Like CDs for photothermal therapy, solar water evaporation and purification technologies have also benefited from substituting chemical precursors with biomass-waste-derived precursors in their fabrication process (Wareing et al., 2021). Biomass-waste-derived CDs are synthesized through green synthesis protocols, which use nonhazardous and benign chemistry protocols that not only produce less toxicity but also bring significant sustainability merits by reducing the overall cost and energy of the production process. Accordingly, Chao et al. (2021) reported the first utilization of biomass-derived CDs in photothermal water evaporation where wood lignin-derived CDs were integrated with dignified natural wood for vapor transportation and *in situ* thermogenesis. Zhou et al. (2021) demonstrated that salt-rejecting solar evaporators with high thermal insulation and light absorption capacity were produced by assembling CDs onto vertically aligned acetate fibers. Their solar water evaporator system demonstrated a significantly high solar-to-vapor efficiency and good thermal cycling stability. Similar results were reported by Zhang et al. (2021), Wang et al. (2022a), and Xu et al. (2022) in which coal pitch-derived CDs with concentration-dependent tunable fluorescence property were packed along the cotton fibers (CFs), cellulose nanofibrils, and potato-derived starch aerogels, respectively, demonstrating durable photothermal solar evaporators for water purification applications.

Zheng et al. incorporated metallic dopants to fabricate a heterogeneous nanocomposite comprising Au-Bi_2_MoO_6_-carbon dot nanoparticles which offered synergistic broadband light absorption and high solar-to-heat conversion efficiency. They showed that integrating three typical photothermal nanoparticles into one ternary nanocomposite can improve light absorption, and PCE due to the formation of heterojunctions between the three primary components. They also demonstrated that their Au-Bi_2_MoO_6_-carbon dot nanocomposites could be used for efficient thermoelectric power generation (Zheng et al., 2020). Likewise, Ginting et al. (2022) engineered a complex system comprising of MWCNTs-ZrO2-Ni CDs brush coated on melamine foam that enables rapid water transport to facilitate solar-driven photothermal water evaporation.

### Photothermal antibacterial activity

The rise of bacterial drug resistance is a significant burden on our global healthcare system, fueled by the high rate of antibiotic use. Therefore, there is a critical need to develop alternate disease mitigation strategies that do not rely on antibiotics. Recently, photothermal nanomaterials, including CDs, have been harnessed for photothermal antibacterial therapy by raising the local temperature at the bacterial site, thus making the bacteria less susceptible to drug resistance by preventing the formation of protective biofilm (Yan et al., 2021). The photothermal process also enhances the ROS generation as the local temperature increase resulting in increased permeability of the bacterial cell wall, which further aids in bacterial cell death (Wen et al., 2022). In a series of reports, heteroatom-doped CDs with excellent fluorescence emission were hybridized with curcumin (Figure 1C), and methylene blue as common photosensitizers (PS), which enhanced of the ROS generation, water solubility of the applied PS, and the photothermal heat generation for bacterial inactivation (Yan et al., 2021; Lv et al., 2022; Wen et al., 2022). Similarly, Wen et al., demonstrated that CDs modified with quaternary ammonium salt can simultaneously provide photothermal antibacterial activity and wound healing without any inflammatory damage to surrounding tissues (Chu et al., 2022). Moniruzzaman et al. prepared polychromatic CDs by changing the acidic strength of the CDs synthesis reaction. Among the prepared CDs, yellow color emissive ones demonstrated a higher PCE due to the presence of polyaromatic sp^2^ domains and abundant oxidized functional groups on their surface. Their yellow-color emissive CDs were applied in the elimination of *Bacillus subtilis* bacteria *via* the eradication of its membrane at high temperatures (Moniruzzaman et al., 2022).

### Miscellaneous photothermal applications

Carbon dots have been utilized as photothermal dopants in photo-responsive shape memory polymer composites (SMPs). Recently, Li et al. (2021c) integrated CDs in carbonized wood to develop a thermal storage phase change material (PEG) with excellent shape stability and increased thermal conductivity. Successively, Yan et al. doped SMPs with biomass-derived carbon nanomaterials produced from the crushed farmland waste of corn straws. The strands of SMP doped with various contents of CDs (thickness 0.2 nm) were first deformed to an “L” shape by applying heat (60°C) and force. Next, The SMPs were exposed to a NIR light source, causing the CDs to heat up to 39°C, which resulted in the recovery of the initial straight shape. The authors claimed that reinforcing SMPs with biomass-derived carbon nanomaterials can open the path to the large-scale production of environmentally friendly and biocompatible smart polymers and intelligent soft robotics (Yan et al., 2022).

Another interesting photothermal application of CDs was demonstrated in a report by Wang et al., (2022b), in which sugar-derived CDs integrated with Bi_2_MoO_6_ nanocrystals were applied for the photothermal self-healing of polydimethylsiloxane (PDMS). The photothermal self-healing property was tested using the scratch and illumination technique in which the surface of a PDMS-Bi_2_MoO_6_-CDs sample was scratched, and its morphological changes were analyzed using a confocal laser scanning microscope. The authors claimed that CDs’ photothermal heat results in the formation and reconstruction of hydrogen bonds in PDMS. This phenomenon can stimulate the movement of PDMS chains, which in turn causes its surface self-healing.

Wan et al. reported the first fabrication of photothermal plastics by covalently immobilizing CDs with photocaged reactivity within the plastic matrix. The prepared photothermal plastics were used for solar-driven thermoelectric energy production. The authors claimed that the functionality of their carbon dot-assisted photothermal plastic can be expanded to solar steam generation and phase change energy storage (Wan et al., 2022).

## Photothermal conversion efficiency of carbon dots

The photothermal applications of CDs and their associated PCE are tabulated in Table 1 with their corresponding synthesis protocol, precursor type, diameter size, nitrogen and oxygen contents, absorption and fluorescence peaks, power, and wavelength of the light source. Upon performing a meta-analysis, we find that the synthesis protocol, choice of precursors, and functionalization are the key parameters determining the PCE of resulting CDs. The efficiency of photothermal conversion in CDs is a direct consequence of the final band gap energy of the fabricated CDs. Thus, any chemical/physical modification that decreases the band-gap energy in CDs can enhance their PCE. This can be achieved through various methods, including but not limited to hyperconjugation and heteroatom doping (especially nitrogen and oxygen). Most of the CDs that have a combination of nitrogen, and oxygen doping (regardless of their amount) (Geng et al., 2020; Zhao et al., 2020; Zhao et al., 2021; Han et al., 2022) demonstrate a higher PCE (>50%) compared to their counterparts lacking even one of these dopants (Wang et al., 2017; Deng et al., 2021; Shu et al., 2021) (PCE < 30%). However, comparing the impact of each controlling factor is limited due to the lack of sufficient data or the absence of relative characterization in available reports. Therefore, a model that considers all parameters known for enhancing the PCE of CDs such as band-gap energy, diameter size, nitrogen and oxygen content and type of nitrogen content (Pyridinic-N and pyrrolic-N) needs to be developed to construct a PCE landscape, whereby researchers can change accessible fabrication parameters to yield the desired application specific PCE in CDs.

**TABLE 1:** Photothermal application, synthesis protocol, properties, and PCE of carbon dots.

Applications	NP/composite system	Precursors	Synthesis	CDs properties					Light source		PCE (η) (%)	Ee (%)	Reference
				d (nm)	N (%)	O (%)	Abs (nm)	Em (nm)	Power/Power Density	Excitation wavelength (nm)			
Combined phototherapy/chemotherapy	Fe3O4@FC	Chemical	Solvothermal	∼4	—	—	260, 390	∼420	1.5 W/cm^2^	700 and 800	—	—	Wang et al. (2014b)
								*λ*_ex_ = 365					
Photothermal cancer therapy	PEGylated CNPs	Chemical	Chemical oxidation	5–10	—	—	365	530	3 W	808	—	—	Tu et al. (2014)
								*λ*_ex_ = 365					
Phototherapy	Fe3O4@C-CDs	Chemical	Solvothermal	∼100^a^	—	—	260–540	400–575	1.5 W/cm^2^	NIR	—	—	Wang et al. (2014a)
								*λ*_ex_ = 365					
Photodynamic/photothermal therapy	C-dot	Chemical	Hydrothermal	6–10	—	—	543, 637	640–680	2 W/cm^2^	635	36.2	—	Ge et al. (2016)
								*λ*_ex_ = 365					
Photothermal cancer therapy	CyCD	Chemical	Solvothermal	2.9 ± 0.5	—	—	783	820	2 W/cm^2^	808	38.7	—	Zheng et al. (2016)
								*λ*_ex_ = 720					
Phototherapy	PLGA-b-PEG−CDs	Chemical	Hydrothermal	∼100^a^	—	—	600–800	670	2 W/cm^2^	671	38.5	—	Hou et al. (2017)
								*λ*_ex_ = 365					
Phototherapy	S. Se-codoped CDs	Chemical	Hydrothermal	∼20	0.58	12.79	526	731,820	2 W/cm^2^	635	58.2	—	Lan et al. (2017)
								*λ*_ex_ = 460					
Photothermal-chemotherapy	CCHNs	Biomass	Hydrothermal	∼65^a^	—	—	363	472	1.5 W/cm^2^	808	25.2	—	Wang et al. (2017)
								*λ*_ex_ = 400					
Photothermal and chemical dual-modal therapeutic agents	GdN@CQDs/GP	Chemical	Hydrothermal	25–146^a^	—	—	300, 500–650	475	2 W/cm^2^	671	—	—	Zhang et al. (2017)
								*λ*_ex_ = 365					
Phototherapy	CD-PDA	Chemical	Microwave-assisted pyrolysis	∼25^a^	—	—	274, 370	450, 500	2 W/cm^2^	808	35	—	Bai et al. (2018)
								*λ*_ex_ = 350, 420					
Phototherapy	PEG-modified CuCD NSs	Chemical	Solvothermal	20–30^a^			300–900^a^	430	2 W/cm^2^	808	39.2	—	Bao et al. (2018)
								*λ*_ex_ = 360					
Photothermal/photodynamic therapies	Cu,N-CDs	Chemical	Hydrothermal	2.5–4.3	9.7	—	366–456	433–513	1 W/cm^2^	808	—	—	Guo et al. (2018)
								*λ*_ex_ = 366–456					
Photodynamic/photothermal therapy	HBCDs	Biomass	Solvothermal	4.76 ± 0.43	4.33	22.19	450, 580	610	0.8 W/cm^2^	635	27.6	—	Jia et al. (2018)
								*λ*_ex_ = 365					
Phototherapy	Carbon dots	Chemical	Hydrothermal	∼5.3	—	—	282, 363, 400	490	2 W/cm^2^	808	52.3	—	Lan et al. (2018)
								*λ*_ex_ = 365					
SWE	CDs@Wood	Waste	Chemical oxidation	—	—	—	Broad 400–2,500	—	1 kW/m^2^	Solar	—	92.5	Hou et al. (2019)
Phototherapy	Carbon dots	Biomass	Hydrothermal	∼6	4.9	14.9	Broad 400–800	400–474, 925	1.4 W/cm^2^	808	30.6	—	Li et al. (2019)
								*λ*_ex_ = 250–400, 808					
Phototherapy	CD@MSN	Chemical	Hydrogen bond/electrostatic-assisted coassembly	∼5	—	—	Broad 200–900	—	2 W/cm^2^	808	—		Qian et al. (2019)
Solar-enabled water remediation	CMC/Chitosan/C-dots hydrogel	Chemical	Solvothermal	8 ± 2 for CDs	—	—	300, 500	∼620	1 kW/m^2^	Solar	—	89	Singh et al. (2019)
								*λ*_ex_ = 510					
Photothermal/photodynamic synergistic cancer therapy	Ce6-RCDs	Chemical	Solvothermal	∼3.7 avg	20.71^b^	7.85^b^	∼540	∼640	2.53 W/cm^2^	671	46	—	Indriyati et al. (2022)
								*λ*_ex_ = 550					
Phototherapy	NIR–II–CDs	Chemical	Nitration, microwave treatment	10–13	21.43	14.24	∼540	∼470	0.6 W/cm^2^	1064	81.3	—	Geng et al. (2020)
								*λ*_ex_ = 370–450					
Water purification and optical switching	C-Dot–CMC–PNIPAM hydrogel	Chemical	Hydrothermal	0.7–2.5	—	—	∼320, 600	∼540	1 kW/m^2^	Solar	—	—	Singh and Jelinek, (2020)
								*λ*_ex_ = 440					
Phototherapy	CuS/Carbon dot nanocomposites	Chemical	Microwave-assisted hydrothermal	∼200^a^	—	—	Broad 500–900	∼460	0.5 W/cm^2^	808	39.7	—	Yu et al. (2020)
								*λ*_ex_ = 365					
Photothermal/photodynamic synergistic cancer therapy	Carbon dots	Chemical	Solvothermal	∼2.5 ± 0.5	3.04	20.2	Broad 400–800	∼575–585	500 mW	800	73.5	—	Zhao et al. (2020)
								*λ*_ex_ = 365					
SWE, desalination, electricity generation	Au@Bi2MoO6-CDs	Chemical	Solvothermal	∼5	—	—	285	—	1 kW/m^2^	Solar	—	97.1	Zheng et al. (2020)
SWE	LCQDs	Biomass	Solvothermal	—	5.81	45.45	Broad 400–2,500	∼460	1 kW/m^2^	Solar	—	79.5	Chao et al. (2021)
								*λ*_ex_ = 365					
Chemo/photothermal synergistic therapy	RCDs	Biomass	Solvothermal	1.0–3.5	4.04	30.07	Broad 200–800	680	1.5 W/cm^2^	680	34	—	Hu et al. (2021)
								*λ*_ex_ = 417					
Phototherapy	S-CDs	Biomass	Hydrothermal	∼3.2	—	35.73	300, 360	440	1.1 W/cm^2^	808	55.4	—	Kim et al. (2021)
								*λ*_ex_ = 360					
Long-term shape-stabilized composite phase change materials with superior thermal energy conversion capacity	Carbon dots	Chemical	Hydrothermal	1.3–4	10.29	16.12	—	—	1 kW/m^2^	Solar	—	84.4	Li et al. (2021c)
Phototherapy	Anti-EpCAM@PDA-CDs@Pt (IV)	Chemical	Microwave treatment	1.7 avg	—	—	275, 360	454	—	808	39.1	—	Li et al. (2021b)
								*λ*_ex_ = 365					
Chemo–photothermal targeted drug delivery	PDA-PEI@N,S-CQDs	Chemical	Solvothermal	2–3	—	—	600–800	∼460	2 W/cm^2^	808	28.5	—	Shu et al. (2021)
								*λ*_ex_ = 430					
Antibacterial activity	CDs/Cur	Chemical	Hydrothermal	∼3.2	6.1	20.9	237, 338.405.560–820	∼450	500 mW/cm^2^	405 + 808	—	—	Yan et al. (2021)
								*λ*_ex_ = 360					
SWE	CFs@CDs	Waste	H_2_O_2_ exfoliation	—	—	—	Broad 600–2,400	∼460	1 kW/m^2^	Solar	—	93.6^a^	Zhang et al. (2021)
								*λ*_ex_ = 365					
Phototherapy	Carbon dots	Chemical	Solvothermal	∼4.4	2.8	22.8	∼355, 550–820	563	2 W/cm^2^	808	54.7	—	Zhao et al. (2021)
								*λ*_ex_ = 520					
SWE	VAAFs@CDs	Waste	H_2_O_2_ exfoliation	∼5	—	—	Broad 400–2,500	450	1 kW/m^2^	Solar	—	93.9	Zhou et al. (2021)
								*λ*_ex_ = 365					
Ammonium salt/photothermal synergistic antibacterial therapy	RCDs-C_35_	Chemical	Carboxyl-amine reaction	∼8.5	—	—	Broad 500–1,000	∼525	2 W/cm^2^	808	35	—	Chu et al. (2022)
								*λ*_ex_ = 380–560					
Phototherapy	supra-CNDs	Chemical	Hydrothermal	10–60	—	—	Broad 450–900	∼450	2 W/cm^2^	808	55.49	—	Duan et al. (2022)
								*λ*_ex_ = 365					
SWE	MWCNTs-ZrO2-Ni@CQDs	Biomass	Hydrothermal	—	—	—	310	∼495	1 kW/m^2^	Solar	—	89.6	Ginting et al. (2022)
								*λ*_ex_ = 365					
Phototherapy/tumor penetration	PCDs	Chemical	Solvothermal	∼5	8.70	8.80	Broad 400–900	∼450, 650	1 W/cm^2^	808	83.72	—	Han et al. (2022)
								*λ*_ex_ = 375, 540					
SWE	CND-FD	Chemical	Microwave treatment	—	—	—	200–350	414	1 kW/m^2^	Solar	—	84	Li et al. (2015)
								*λ*_ex_ = 365					
Photothermal bactericidal efficacy	Y-CQDs	Chemical	Thermal heating^a^	6–10	—	—	305	550	1 W/cm^2^	808	32.6 ± 1	—	Moniruzzaman et al. (2022)
								*λ*_ex_ = 365					
Phototherapy	GR-CDs	Chemical	Top-down acid treatment (Li et al., 2015)	2.6 ± 0.5	—	45.8	230, 265	540	2.5 W/cm^2^	808	45.7	—	Shi et al. (2022)
								*λ*_ex_ = 460					
Tumor-specific and photothermal-augmented chemodynamic therapy	Fc-CD	Chemical	Solvothermal	11.6	—	—	∼300, ∼550	650	0.6 W/cm^2^	660	41.39	—	Sun et al. (2022)
								*λ*_ex_ = 550					
Photothermal plastics	P-CDs	Chemical	Solvothermal	∼3.7 avg	2.07	22.76	350	∼490, ∼540	100 mW/cm^2^	Solar	33.2	—	Wan et al. (2022)
								*λ*_ex_ = 375					
Solar-driven water purification	CNFs@CDs	Waste	Selective oxidation	—	—	—	Broad 500–2,500	—	1 kW/m^2^	Solar	—	96.45	Wang et al. (2022a)
Antibacterial therapy	CDs-Cur	Chemical	Microwave treatment	6–8	—	—	360	460	200 mW/cm^2^	405 + 808	—	—	Wen et al. (2022)
								*λ*_ex_ = 360					
SWE	C-CDSA	Biomass	H_2_O_2_ exfoliation	—	—	—	Broad 300–2,400	—	1 kW/m^2^	Solar	—	93.5	Xu et al. (2022)
Photo-responsive shape memory polymer	BCMs	Biomass	Pyrolysis followed by acid oxidation treatment	—	—	—	—	—	1.90 W/cm^2^	808	—	—	Yan et al. (2022)
SWE	CQDs	Chemical	Microwave Treatment	13 avg	—	—	275	450	2.5 kW/m^2^	Solar	69.89	—	Zhou et al. (2022)
								*λ*_ex_ = 365					

^a^ Value represents the diameter of the photothermal nanoparticle-composite system instead of carbon dots.

^b^ Calculated based on the reported molar ratio.

## Conclusion and future perspectives

The attractive photothermal performance of CDs in multiple applications promises their continuous development in the upcoming years. Photothermal conversion in metallic nanoparticles has benefitted tremendously from a mechanistic understanding and computational modeling of the underlying localized surface plasmonic resonance (LSPR) phenomenon. Unlike metallic nanoparticles, there is very little mechanistic understanding of photothermal conversion in carbon-based nanoparticles. Such insight can accelerate the application of CDs for photothermal applications. Furthermore, substituting chemical precursors with heteroatom-rich biomass and waste precursors for the fabrication of CDs can not only introduce required heteroatoms such as S, N, and O into the structure of these CDs, but also increase their biocompatibility, ease-of-preparation, and cost-effectivity. Exploring other photothermal applications in which CDs have not yet been utilized, such as photothermal laser ignition (Hong et al., 2022), photothermal nanoreactors (Li et al., 2021d), and solar collector (Sun et al., 2019) can expand their application space.
